# Neural Alterations in Acquired Age-Related Hearing Loss

**DOI:** 10.3389/fpsyg.2016.00828

**Published:** 2016-06-02

**Authors:** Raksha A. Mudar, Fatima T. Husain

**Affiliations:** ^1^Department of Speech and Hearing Science, University of Illinois at Urbana–Champaign, ChampaignIL, USA; ^2^Neuroscience Program, University of Illinois at Urbana–Champaign, ChampaignIL, USA; ^3^Beckman Institute for Advanced Science and Technology, University of Illinois at Urbana–Champaign, ChampaignIL, USA

**Keywords:** hearing loss, aging, dementia, neural, cognition, MRI, EEG

## Abstract

Hearing loss is one of the most prevalent chronic health conditions in older adults. Growing evidence suggests that hearing loss is associated with reduced cognitive functioning and incident dementia. In this mini-review, we briefly examine literature on anatomical and functional alterations in the brains of adults with acquired age-associated hearing loss, which may underlie the cognitive consequences observed in this population, focusing on studies that have used structural and functional magnetic resonance imaging, diffusion tensor imaging, and event-related electroencephalography. We discuss structural and functional alterations observed in the temporal and frontal cortices and the limbic system. These neural alterations are discussed in the context of common cause, information-degradation, and sensory-deprivation hypotheses, and we suggest possible rehabilitation strategies. Although, we are beginning to learn more about changes in neural architecture and functionality related to age-associated hearing loss, much work remains to be done. Understanding the neural alterations will provide objective markers for early identification of neural consequences of age-associated hearing loss and for evaluating benefits of intervention approaches.

## Introduction

Hearing loss is the third most prevalent chronic condition affecting older adults (Cruickshanks et al., 1998) and is one of the leading causes of years lived with disability worldwide (Mathers et al., 2003). The incidence and prevalence of hearing loss increases with age (Yueh et al., 2003; Feder et al., 2015), similar to other chronic conditions, such as dementia. In the United States, nearly two-thirds of individuals 70 years and older have disabling age-associated hearing loss (Lin et al., 2011c) with comparable trends reported around the globe (Roth et al., 2011; Feder et al., 2015). Only a small proportion of these older adults who would potentially benefit from treatment seek help (Lin et al., 2011c; Feder et al., 2015); the rest are at risk of suffering serious psychosocial and cognitive consequences.

Decades of research have characterized the impact of varying degrees of hearing loss on auditory processing, speech communication, and psychosocial well-being (e.g., Strawbridge et al., 2000; Heine and Browning, 2002; Kramer et al., 2002; Dalton et al., 2003; Kamil and Lin, 2015). The detrimental impact of hearing loss on cognition has more recently come to light (Bernabei et al., 2014; Martini et al., 2014; Wingfield and Peelle, 2015). Data from large population-based longitudinal studies, which have characterized hearing ability using audiometric assessments, suggest that hearing loss is independently associated with poorer cognitive functioning (Valentijn et al., 2005; Tay et al., 2006; Lin et al., 2011a) and incident dementia (Uhlmann et al., 1989; Lin et al., 2011b, 2013; Gurgel et al., 2014) when confounding factors such as age and sex are controlled. For instance, Lin et al. (2011a) found that a 25 dB hearing loss (average of hearing thresholds at 0.5, 1, 2, and 4 kHz in the better hearing ear) results in cognitive performance equivalent to 6.8 years below what is appropriate for the subjects’ chronological age. Furthermore, there is a positive correlation between degree of hearing loss and dementia risk. Individuals with mild hearing loss are twice as likely, and those with moderate hearing loss are three times as likely, to develop dementia compared to those with normal hearing (Lin et al., 2011b).

Several hypotheses linking age-related hearing loss and cognitive decline have been proposed, but a cause-effect link has yet to be established (Wayne and Johnsrude, 2015). Some have proposed that cognitive decline and age-related hearing loss are expressions of widespread neural degeneration that occurs during aging, an idea referred to as the *common cause* hypothesis (Lindenberger and Baltes, 1994; Anstey et al., 2001). However, a study by Humes et al. (2013) suggests an association between global sensory processing and cognitive processing independent of age. In line with these findings, some researchers have suggested that hearing loss results in compromised cognitive performance and functional decline because cognitive resources are increasingly dedicated to auditory processing to the detriment of other processes (e.g., executive function). These cognitive consequences are considered to be instantaneous per the *information-degradation* hypothesis (Lindenberger and Baltes, 1994; Schneider and Pichora-Fuller, 2000; Pichora-Fuller, 2003; Tun et al., 2009) or long-term with changes in brain plasticity per the *sensory-deprivation* hypothesis (Lindenberger and Baltes, 1994; Schneider and Pichora-Fuller, 2000; Pichora-Fuller, 2003; Humes et al., 2013; Lin et al., 2013). Regardless of the theoretical context, numerous studies have documented cognitive decline in individuals with hearing loss in both auditory (e.g., verbal memory) and non-auditory cognitive tasks (e.g., Digit Symbol Substitution Test, Trails B; e.g., Uhlmann et al., 1989; Li and Lindenberger, 2002; Gussekloo et al., 2005; Helzner et al., 2005; Valentijn et al., 2005; Tay et al., 2006; Humes et al., 2010; Lin et al., 2011a, 2013; Rönnberg et al., 2011; Heyl and Wahl, 2012), suggesting that effects of age-associated hearing loss on cognition are central in origin. Understanding whether these cognitive declines are driven by additional neuroplastic changes above and beyond those that occur in aging will have both theoretical and clinical implications. Given that hearing loss is more amenable to remediation compared to other risk factors associated with dementia, alleviating this risk is beneficial. Understanding the impact of age-associated hearing loss on neural architecture and functionality will enable researchers to assess the efficacy of such remediation approaches and determine whether their effects are permanent (Lin et al., 2013).

In this mini-review, we briefly examine literature on anatomical and functional alterations in the brains of adults with acquired age-associated hearing loss, which may underlie the cognitive consequences observed in this population. Based on this literature, we evaluate the support for existing hypotheses related to hearing loss and cognition, and suggest approaches to remediate age-associated hearing loss to prevent cognitive deterioration.

## Anatomical Changes Observed in Age-Associated Hearing Loss

The most commonly used techniques to measure anatomical changes in the brain include MRI voxel-based morphometry (VBM) and diffusion tensor imaging (DTI). VBM has been used to investigate changes in gray matter thickness, concentration, and volume, with declines in gray matter observed in a number of chronic conditions such as depression (Grieve et al., 2013) and dementia (Busatto et al., 2008). DTI has been used to estimate the integrity of white matter tracts by examining the diffusion of water molecules (Basser and Pierpaoli, 1996). The most commonly used measure of tract integrity is fractional anisotropy (FA), which ranges in value from 0 to 1. The higher the FA value, the healthier is the extent of myelination and fiber density.

Using VBM, Peelle et al. (2011) examined whether normal variations in hearing ability (average threshold in better ear at 1 kHz, 2 kHz, and 4 kHz) affect brain structures that support speech comprehension in older adults. Poorer hearing ability was significantly associated with reduced gray matter volume in the right auditory cortex with a similar, non-significant trend in the left auditory cortex, implying a link between hearing ability and structural integrity of the auditory cortex. A later study by Eckert et al. (2012) parsed the impact of low and high frequency hearing loss on auditory cortex morphology in older adults. The components related to low (primarily < 2 kHz) and high (≥2 kHz) frequencies were derived using factor analysis of audiometric thresholds. Reductions in gray matter volume in the bilateral primary auditory cortex correlated with high frequency, age-related hearing loss even when age-related structural changes were controlled. Other VBM studies have demonstrated that structural alterations in individuals with hearing loss are not restricted to the auditory cortex. Wong et al. (2010) found that older adults exhibited declines in prefrontal cortex gray matter volume and thickness that correlated with poorer ability to perceive speech in noise (QuickSIN test at 0 dB signal-to-noise ratio), even when raw brain volume changes due to age were considered. No such relationship was observed in younger adults. Note that the older group had lower hearing sensitivity compared to the younger group at higher frequencies (6 kHz and 8 kHz).

The studies by Wong et al. (2010), Peelle et al. (2011), and Eckert et al. (2012) are crucial in bringing to light the gray matter decline in older adults with hearing loss. Although these studies statistically controlled age effects in their analyses, they lacked an age-matched normal hearing control group, thus requiring further validation of these findings. This gap was filled by a cross-sectional study conducted by Husain et al. (2011) that examined changes in gray matter using VBM in individuals with mild-to-moderate hearing loss (<70 dB HL threshold 4–8 kHz) and normal hearing age- and gender-matched controls (<25 dB HL between 0.25–8 kHz). The researchers found declines in gray matter volume in the right anterior cingulate and bilateral medial frontal gyrus via whole-brain analysis and in the superior temporal cortex in a region-of-interest analysis in the hearing loss group compared to the controls. These results suggest that gray matter reductions observed in the brains of older adults with hearing loss are not related to age alone, supporting the findings of the other VBM studies discussed. However, caution is warranted in interpreting these results because studies reviewed thus far are cross-sectional and it is unknown whether these differences occurred before or after hearing loss onset.

A more recent longitudinal study (Lin et al., 2014) offers additional support for a link between peripheral hearing impairment and accelerated brain atrophy independent of age. Lin et al. (2014) measured changes in brain volume in older adults with hearing loss (speech-frequency pure tone average >25 dB) as compared to normal hearing controls over a 6-year follow-up period. They observed that individuals with hearing loss showed accelerated decline in whole brain volume and regional decline in the right temporal lobe. Beyond these studies of acquired hearing loss, studies involving deaf individuals have shown profound structural changes in the brain (Shibata, 2007; Pénicaud et al., 2013; Hribar et al., 2014; Olulade et al., 2014). Some of these studies have also observed neuroplastic changes in gray matter due to compensatory behavior (Pénicaud et al., 2013; Olulade et al., 2014), which suggests that gray matter changes observed in age-associated hearing loss may be similarly amenable to actively learned compensatory behaviors guided by interventions.

Hearing loss appears to be a prominent trigger for changes in white matter (Lee et al., 2004; Lin et al., 2008; Kim et al., 2009; Husain et al., 2011; Rachakonda et al., 2014). DTI studies of participants with hearing loss (Chang et al., 2004; Lin et al., 2008; Husain et al., 2011) have found reduced FA values (indicative of more diffuse transmission) in several white matter pathways leading into and out of the auditory cortex, suggesting changes in microstructural integrity of tracts. However, the population in these studies have mostly involved young to middle-aged adults (Husain et al., 2011). In contrast, Profant et al. (2014) compared normal hearing young adults and two groups of elderly with mild hearing loss, with increased thresholds beginning at 1 kHz or 2 KHz, respectively. In line with the findings of other DTI studies but using a different metric of directional diffusivity, these researchers noted differential trends in the orientation of tracts connecting the inferior colliculus to the primary auditory cortex in the elderly groups, but the threshold differences between the two elderly groups did not have an effect.

In general, studies have demonstrated reduced cortical volumes in the auditory processing areas in the superior temporal cortex and declines in white matter tract integrity of central auditory pathways, with some exceptions (Profant et al., 2014), in individuals with hearing loss. Regions beyond the canonical auditory processing areas, such as the frontal cortices, also exhibit effects of hearing loss. The findings suggest that the structural alterations observed in older adults with hearing loss cannot be fully explained by mechanisms related to age alone, providing credence to the information degradation and sensory deprivation hypotheses. It remains to be seen if such anatomical changes are reversible with intervention.

## Functional Changes Observed in Age-Associated Hearing Loss

A handful of studies have examined functional brain changes in age-associated hearing loss. Here, we focus on studies that have used functional magnetic resonance imaging (fMRI), which measures brain activity by detecting changes associated with blood flow, and event-related potentials (ERPs), which measure electrical activity in the brain that is time locked to a cognitive process. The fMRI studies in particular have varied widely in terms of the stimuli used (e.g., simple acoustic stimuli, affective pictures) and the paradigms employed. Thus, our primary goal is to summarize whether these fMRI studies have observed (i) any relationship between functional brain activation patterns and hearing ability or (ii) any differences in brain activation patterns in individuals with age-associated hearing loss as compared to normal hearing controls.

Peelle et al. (2011) used fMRI to examine the relationship between brain functions in older adults during sentence processing and hearing ability (average threshold in better ear at 1 kHz, 2 kHz, and 4 kHz). They found that differences in hearing ability predicted the degree of neural recruitment in the bilateral superior temporal gyri, thalamus, and brainstem during sentence comprehension. In particular, poorer hearing listeners showed less language-driven brain activity even when age was controlled. In another fMRI study, Profant et al. (2015) used pink noise to study the neural response in two groups of older adults with mild hearing loss, with increased thresholds beginning at 1 kHz or 4 KHz, respectively, and normal hearing young controls. Older adults with hearing loss showed greater response to acoustical stimuli in the temporal lobes in comparison with young subjects. Additionally, older adults showed more pronounced activation in the right temporal lobe compared to activation in left temporal lobe, whereas the young control group showed left lateralization. While these findings demonstrate noticeable alterations in brain function in age-associated hearing loss, the extent to which these alterations are driven by age and/or hearing loss cannot be determined because these studies lacked age-matched normal hearing and hearing impaired younger control groups.

Chen et al. (2016) similarly used fMRI to examine the effect of hearing loss on engagement of the auditory cortex during the processing of monosyllabic words in older adults with hearing loss (26–40 dB average hearing threshold from 0.5–2 kHz) compared to age-matched and young normal hearing controls. Bilateral activation in the auditory cortex was reduced in the hearing loss group compared to the age-matched control group with both right and left ear stimulation, but the differences were not significant. Furthermore, when unilateral sound presentation was used, both older groups showed higher left auditory cortex activation with left ear stimulation, whereas, with right ear stimulation, both older groups showed reduced right auditory cortex activation and bilateral activation compared to the young controls. There were no specific effects of hearing loss. Thus, it appears that aging, rather than hearing loss, had a greater effect on engagement of the auditory cortices.

The fMRI studies discussed above investigated age-associated hearing loss using auditory tasks, whereas Husain et al. (2014) examined the effects of hearing loss on emotional processing using task- and rest-based fMRI in middle aged individuals with bilateral high-frequency hearing loss (pure-tone thresholds 30–70 dB HL for 4–8 kHz) using an age matched-control group with normal hearing. The task used required participants to rate affective stimuli from the International Affective Digital Sounds database (Bradley and Lang, 2007) as pleasant, unpleasant, or neutral. For the resting state fMRI, participants were told to fixate on a “+” sign on the screen for 5 min. They found that the normal hearing group employed the expected limbic and auditory regions to a greater extent when processing affective stimuli compared to participants with hearing loss. Additionally, resting-based fMRI showed alterations in the dorsal attention network and default mode network in the hearing loss participants compared to controls. These results not only offer initial evidence supporting functional alterations beyond the auditory cortex in individuals with hearing loss but suggest that these alterations are not related to aging alone.

Similar to Husain et al. (2014), two ERP studies have demonstrated functional alterations in individuals with hearing loss beyond the auditory cortices. Campbell and Sharma (2013) examined auditory evoked potentials (AEP) in participants with bilateral mild-to-moderate high frequency (2–8 kHz) hearing loss and normal hearing controls. The auditory stimuli involved the speech syllable /ba/. Individuals with hearing loss showed increases in latency and amplitude of the P2 AEP relative to control subjects. Also, behavioral performance on a speech in noise test (QuickSIN) was significantly correlated with increases in P2 latency. Cortical source localization revealed decreased activation in the temporal cortex and increased activation in frontal cortical areas in individuals with hearing loss compared to controls, suggesting potential changes in the allocation of cortical resources.

In a subsequent study, Campbell and Sharma (2014) examined visual evoked potentials (VEP) in middle-aged adults with bilateral mild-to-moderate high frequency (2–8 kHz) hearing loss and normal hearing controls. The visual stimuli involved sinusoidal concentric grating that morphed into circle and star figures. The amplitudes of P1, N1, and P2 VEP were significantly larger in individuals with hearing loss. Additionally, N1 latency was decreased in individuals with hearing loss compared to controls and correlated with speech perception performance in noise. Cortical source localization revealed increased activation of auditory-processing temporal areas with visual stimulation in hearing-impaired adults suggesting visual cross-modal re-organization.

In summary, there appear to be functional alterations in the engagement of auditory cortex in age-associated hearing impairment, especially during processing of sounds. Furthermore, emerging evidence suggests that these functional alterations are noticeable in other brain regions including frontal cortices and the limbic system. These results suggest that aging may be a major factor related to changes in the functioning of the auditory system, supporting the common cause hypothesis. However, functional alterations observed in the non-auditory regions lend support to information degradation and sensory deprivation theories, indicating that these frameworks combined might provide a better explanation of age-associated hearing loss (Wayne and Johnsrude, 2015).

## Summary and Future Directions

Estimates suggest that 35.6 million people lived with dementia worldwide in 2010 (Prince et al., 2013). A sharp increase in this number is anticipated in the next few decades because of the shifting age profiles of the global population. Delaying, if not preventing, onset of cognitive decline in individuals with age-associated hearing loss may have a significant impact on dementia prevention. Understanding the neural alterations associated with hearing loss will provide objective markers for early identification of neural consequences of age-associated hearing loss and for evaluating benefits of interventions.

Existing evidence indicates that there are anatomical and functional alterations in the brains of individuals with age-associated hearing loss that are independent of age effects. These changes appear to occur not only in the auditory processing regions, but also in regions related to attention and emotional processing. Future neuroimaging and neurophysiological studies involving audiometrically and cognitively well-characterized participant pools are necessary to further identify changes in neural architecture and functionality related to hearing loss. As evidence starts accumulating, current theories linking aging and hearing loss should be updated to reflect new data.

**FIGURE 1:**
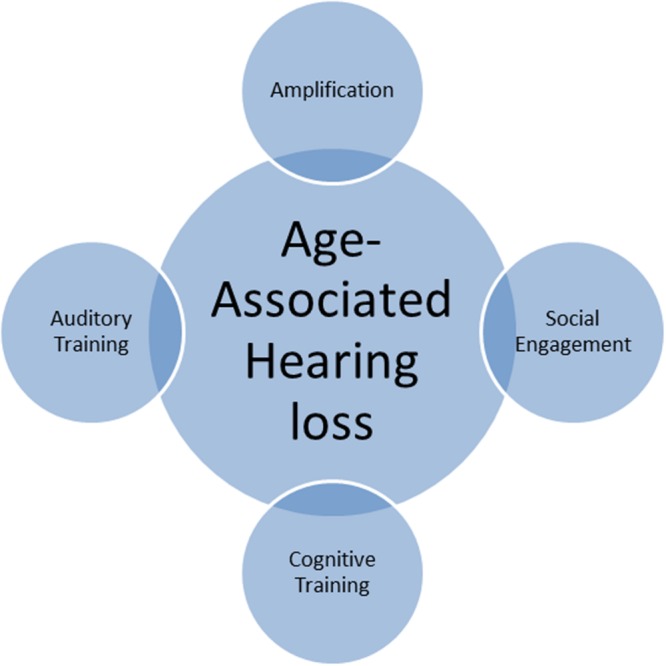
**Age-associated hearing loss: Rehabilitation strategies**.

From the public health standpoint, a critical question related to the cognitive and neural consequences observed in age-associated hearing loss is whether these changes can be reversed with interventions, in turn mitigating dementia risk. A handful of studies have suggested that correction of hearing loss with amplification (Appollonio et al., 1996; Cacciatore et al., 1999; Amieva et al., 2015) and cochlear implants (CI; Mosnier et al., 2015) has positive effects on cognition. However, the current evidence for reversibility of cognitive decline with amplification/CI is still meager. Several studies have observed no positive benefits of amplification on cognition (Valentijn et al., 2005; van Hooren et al., 2005; Lin et al., 2013), indicating that amplification alone may not be enough, and a more holistic approach to intervention may be warranted (see **Figure 1**).

Auditory training, which involves training individuals to listen to auditory input with active engagement, is another promising approach to intervention (Humes et al., 2014) when used individually or in combination with amplification. In a systematic review, Henshaw and Ferguson (2013) found generalized improvement in speech intelligibility with such training. More recently, they reported improvements in measures of self-reported hearing, competing speech, and executive functions following auditory training (Ferguson and Henshaw, 2015). Additionally, cognitive training involving auditory and non-auditory modalities may also be useful in tackling cognitive and neural consequences of hearing loss. Several review studies have pointed to the efficacy of cognitive training in improving memory, executive function, and attention in typically aging older adults (e.g., Tardif and Simard, 2011; Reijnders et al., 2013) and individuals with mild cognitive impairment (e.g., Gates et al., 2011; Li et al., 2011; Reijnders et al., 2013), and might similarly benefit individuals with age-associated hearing loss. Cognitive training delivered in group session format may also facilitate social engagement and address issues related to depression and social isolation. Furthermore, combined interventions may yield maximal cognitive and psychosocial benefits in this population.

## Author Contributions

RM and FH have both contributed equally to the conception, drafting, and final approval of this manuscript and both agree to be accountable to all aspects of the work in ensuring that questions related to the accuracy or integrity of any part of the work are appropriately investigated and resolved.

## Conflict of Interest Statement

The authors declare that the research was conducted in the absence of any commercial or financial relationships that could be construed as a potential conflict of interest.

The reviewer BM and handling Editor declared their shared affiliation, and the handling Editor states that the process nevertheless met the standards of a fair and objective review.
